# Requirement of the acyl-CoA carrier ACBD6 in myristoylation of proteins: Activation by ligand binding and protein interaction

**DOI:** 10.1371/journal.pone.0229718

**Published:** 2020-02-27

**Authors:** Eric Soupene, Ulrich A. Schatz, Sabine Rudnik-Schöneborn, Frans A. Kuypers

**Affiliations:** 1 Children’s Hospital Oakland Research Institute, Oakland, California, United States of America; 2 Institute of Human Genetics, Medical University Innsbruck, Innsbruck, Austria; Karl-Franzens-Universitat Graz, AUSTRIA

## Abstract

Glycine N-myristoylation is an essential acylation modification modulating the functions, stability, and membrane association of diverse cytosolic proteins in human cells. Myristoyl-CoA is the 14-carbon acyl donor of the acyltransferase reaction. Acyl-CoAs of a chain length compatible with the binding site of the N-myristoyltransferase enzymes (NMT) are competitive inhibitors, and the mechanism protecting these enzymes from unwanted acyl-CoA species requires the acyl-CoA binding protein ACBD6. The acyl-CoA binding domain (ACB) and the ankyrin-repeat motifs (ANK) of ACBD6 can perform their functions independently. Interaction of ANK with human NMT2 was necessary and sufficient to provide protection. Fusion of the ANK module to the acyl-CoA binding protein ACBD1 was sufficient to confer the NMT-stimulatory property of ACBD6 to the chimera. The ACB domain is dispensable and sequestration of the competitor was not the basis for NMT2 protection. Acyl-CoAs bound to ACB modulate the function of the ANK module and act as positive effector of the allosteric activation of the enzyme. The functional relevance of homozygous mutations in *ACBD6* gene, which have not been associated with a disease so far, is presented. Skin-derived fibroblasts of two unrelated individuals with neurodevelopmental disorder and carrying loss of function mutations in the *ACBD6* gene were deficient in protein N-myristoylation. These cells were sensitive to substrate analog competing for myristoyl-CoA binding to NMT. These findings account for the requirement of an ANK-containing acyl-CoA binding protein in the cellular mechanism protecting the NMT enzymes and establish that in human cells, ACBD6 supports the N-myristoylation of proteins.

## Introduction

Myristoylation of proteins is the irreversible covalent attachment of the C14 tail of myristoyl-CoA to an N-terminal glycine. N-myristoylation occurs mainly during translation of nascent peptides, following the removal of the initiator methionine by methionyl aminopeptidase, and exposure of a glycine [1–7]. The activity of the human N-myristoyltransferase enzymes (NMT1 and NMT2) is essential for protein function. N-myristoylation is also crucial for the intracellular development of pathogens such as parasitic protozoa, fungi, and viruses [8–12] and proteins of intra-cellular bacteria can be target of the host NMT enzymes [13].

The hydrophobicity of the C14 tail is not strong enough to force membrane association of myristoylated-proteins [3,6,14]. This property makes it possible for the optional membrane location of myristoylated-proteins and in many instances a distribution switch from cytosol-to-membrane and membrane-to-cytosol is under tight regulation. Some myristoylated-proteins bury the acyl chain in a hydrophobic pocket and maintain a cytosolic presence. The tail can become exposed to membranes following a conformational alteration induced by diverse factors. The reversible membrane association property of myristoylated-proteins is essential for signal transduction and the integration of diverse cellular inputs. Change in location can affect the functions of the myristoylated-proteins as well as of the cytosolic and membrane proteins interacting with them [1,3,4,6,7,15]. The switch from cytosol-to-membrane can be reinforced by polybasic motifs brought to lipid proximity, additional acylation process such as palmitoylation, and interaction with membrane proteins. The membrane-to-cytosol switch can be triggered by phosphorylation, cofactor binding and attack by proteases. An electrostatic switch induced by phosphorylation weakens interaction of a basic cluster to the membrane and results in the release of myristoylated-MARCKS in the cytosol [16]. The Calcium-myristoylation switch of recoverin (unbound/ Ca^2+^ bound) [14,17] and the GTP-myristoylation switch of ARF GTPase (GDP/GTP exchange) trigger exposure of the myristoyl tail and membranes association [18]. Myristoylated proteins can be targeted by proteases liberating soluble peptide of altered function [19] and proteolytic products of non-myristoylated proteins can become myristoylated following the exposure of an internal glycine within a myristoylation recognition motif [20]. In addition, NMT enzymes of infected cells can target viral and bacterial proteins for myristoylation [5]. Conversely, the IpaJ protease of *Shigella flexneri* cleaves the myristoylated-glycine of the host proteins, such as Golgi-associated ARF/ARL family GTPases [21,22].

The 14-carbon chain of myristoyl-CoA is the acyl-donor of the NMT acyl-transferase reaction but the binding site of NMT proteins can accommodate a few other acyl chains [8,23–27]. The NMT enzymes display a binding affinity for myristoyl-CoA (≈600 nM) that is significantly higher than its cellular concentration, ranging from 5 to 200 nM [1,4,15,28]. In the absence of a mechanism protecting NMT from competitive inhibition, the greater abundance of other acyl-CoA, such as C_16_-CoA, would prevent access of the substrate. The human acyl-CoA binding protein ACBD6 interacts with the human (NMT1 and NMT2) and the protozoan *Plasmodium falciparum* NMT enzymes and, protects their activity [29,30]. The *P*. *falciparum* ACBD6 protein also regulates activity of *Pf*NMT [30]. In the NMT2 / ACBD6 complex, the acyl-CoA carrier allows C_14_-CoA to reach NMT binding site, even in the presence of excess concentration of C_16_-CoA and C_12_-CoA [29,30]. The binding activity and the NMT-stimulatory properties of the acyl-CoA binding (ACB) domain and of the ANK-repeat motifs (ANK module) of ACBD6 were determined in this study. The requirement and role of the acyl-CoA ligand bound to the ACB domain in the allosteric activation of the enzyme was established. Human fibroblast deficient for ACBD6 were obtained. These cells were sensitive to the presence of NMT substrate competitor and were deficient in protein myristoylation *in vivo*. We propose that in human cells, ACBD6 is required to support N-myristoylation of proteins and that the active form of the NMT enzyme is defined by an ACBD6 / NMT heterocomplex.

## Methods

### Genetic analyses

Blood collection and skin biopsy were performed with patient #2 and his brother (only blood collection) at the Innsbruck Institute of Human Genetics (Innsbruck, Austria) following ethical committee approval by the Institutional Review Board of the Medical University of Innsbruck (Innsbruck, Austria) and informed consent of the patients’ parents. The study complied with the principles set out in the World Medical Association’s of the Declaration of Helsinki. Patient #2 and his only brother were sons of healthy related parents (first cousins once removed) from Turkey. Both patients have moderate intellectual disability. Exomes were enriched in solution with SureSelectHuman All Exon Kit 60 Mb V6 (Agilent, Santa Clara, CA, USA). DNA fragments were sequenced as 100 bp paired-end reads on an IlluminaHiSeq4000 system (Illumina, San Diego,CA, USA). The analysis was carried out considering the inheritance trait and the minor allele frequency (MAF) of <1% in approx. 12,000 exomes in house. The data analysis pipeline has been described in [31]. Sanger sequencing was performed according to standard protocols.

### Cell culture and growth experiments

Skin-derived fibroblasts of patient #1 and of a non-carrier sibling (provided by Dr. Reza Maroofian) and of patient #2 were grown in high-glucose DMEM supplemented with 10% fetal bovine serum, 2mM glutamine, 5mM non-essential amino acids and 1% (v/v) MEM vitamins (ThermoFisherScientific). Growth measurements were performed in 96-well plates and quantified by staining with the sulforhodamine B (SRB) dye (Sigma). Cells were fixed with 10% ice-cold trichloroacetic acid for 1 hour at 4°C, washed 4 times with water, and air-dried overnight at room temperature. Protein content was stained with 50μl of 0.04% SRB made in 1% acetic acid for 1 hour. Excess dye was removed by four washes with 1% acetic acid, and wells were air-dried for ≥2 hours. Stained proteins were suspended in 50μl of 10mM Tris base for 10 min. Absorbance was measured at 560nm with a microplate reader.

### Myristic acid and 2-hydroxy myristic acid treatments

The effect of myristic acid (Myr) and of 2-hydroxy myristic acid (2-OH Myr) on the growth of the human fibroblast lines were determined in 96-well plates by staining with the SRB dye. Myr (Sigma) and 2-OH Myr (Cayman Chemical) were dissolved in 100% EtOH at a concentration of 100mM. The cells were seeded in 4 wells at about 350 per well and experiments were performed twice for each cell line and treatment condition. The cells were incubated for 24 hours in absence of the drug. At day-0 of the treatment experiment, the medium was replaced with a fresh solution in the absence or the presence of the drug at a concentration of 10-20-40-60-80 and 100μM. Medium was replaced every 48 hours with freshly diluted drugs and one set of plate was fixed and SRB-stained at day-2, day-3, day-4, and day-6.

### Transfection, cell fractionation and myristoylated GFP detection

The pmyrGFP plasmid expressing the Green Fluorescence protein (GFP) fused to the Src myristoylation signal at its N-terminal extremity (myrGFP) under the CMV promoter was obtained from Addgene (#50528; deposited by the Kenneth Yamada Lab). Cells were seeded at a density of 120,000/well in 6-well plate and grown for 24 hours prior to transfection. Cells were transfected with TurboFect (ThermoFisherScientific) and grown for 48 hours in the absence (2 wells) or the presence (4 wells) of 100μM 2-OH Myr. Cells were collected by trypsinization, washed in PBS and suspended in 200μl Tris-HCl 50mM pH 7.4, 100mM sodium chloride, 5mM EDTA and the Halt^™^ Protease Inhibitor Cocktail. Cells were lyzed with a Dounce homogenizer with a tight pestle. The lysate was clarified by centrifugation at 12,000g for 10 minutes at 4°C. The membrane and soluble fractions were obtained by high-speed centrifugation at 120,000g for 1 hour at 4°C. Proteins were separated on denaturing SDS-PAGE 12%, transferred to PVDF membrane and myrGFP and the membrane marker Na+/K+-ATPase α1 were detected with mouse monoclonal antibodies (clone GF28A from ThermoFisherScientific and C464.6 from Santa Cruz Biotechnology, respectively). Two independent transfection experiments were performed for each of the cell lines and immuno-detection was performed in triplicate.

### Protein production and N-myristoyltransferase activity measurements

**Fig 1:**
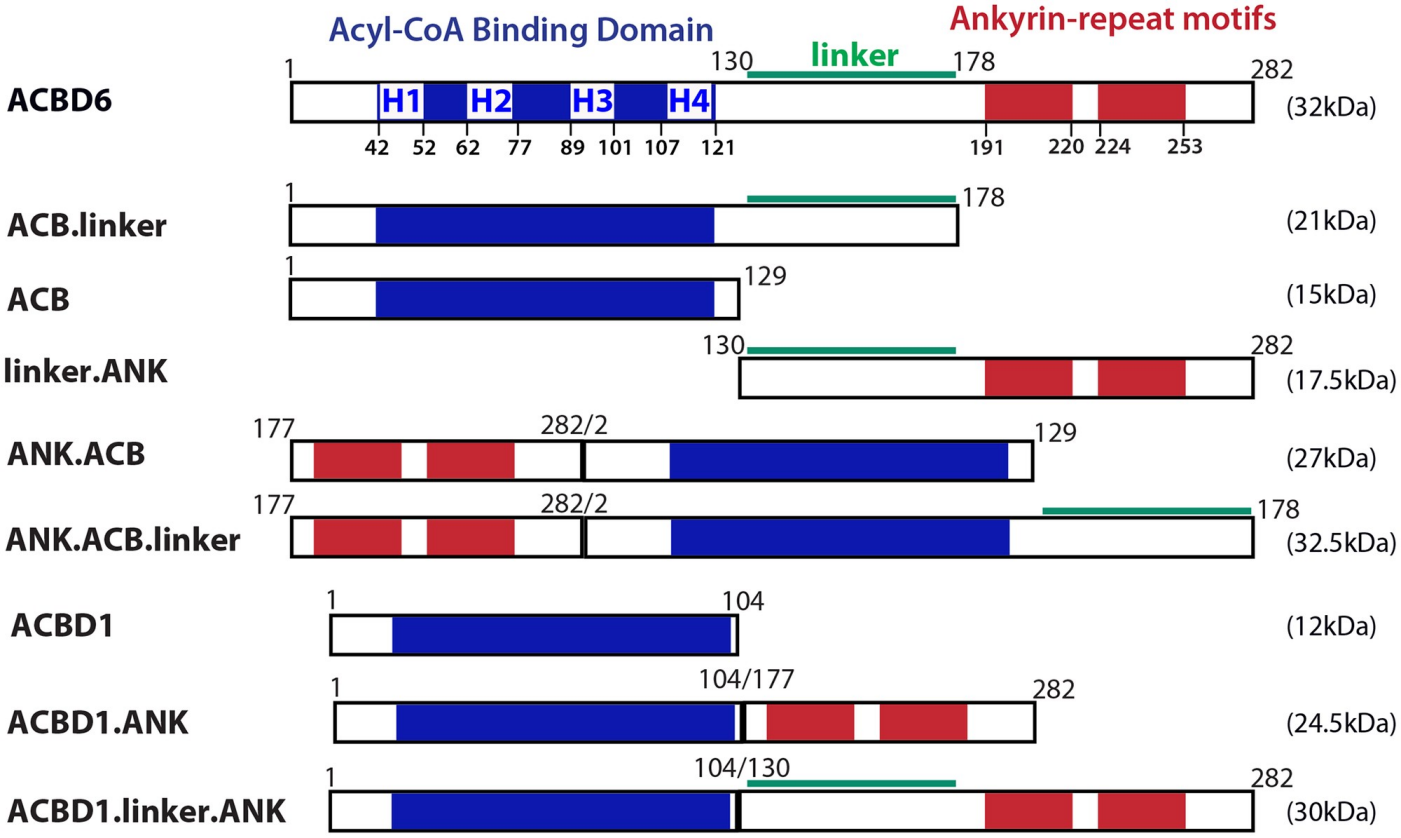
Cartoon representation of the constructs used in this study. The 4 α-helix (H1 to H4) of the acyl-CoA binding domain (blue), of the linker region (green), and of the two ankyrin-repeat motifs (red) of human ACBD6 (282 residues) are shown. For clarity, the different constructs are designed by the presence of the 3 regions: ACB domain (residues 1 to 129), linker (130 to 178), and ANK motifs (177 to 282). All constructs were produced with a hexahistidine tag at their carboxyl-terminal end and the calculated mass (kDa) of each protein is shown on the right. ACBD1 refers to isoform 1 of the product of the *DBI* gene.

The constructs were cloned in pET28a vector and produced with a carboxyl-terminal hexahistidine tag, as previously described [30]. Details of the various truncations and chimeras are presented in Fig 1. DSS (disuccinimidyl suberate; ThermoFisherScientific) crosslinking experiments were performed as previously described [29]. NMT2 (10μM) and the linker.ANK (30μM) recombinant proteins were mixed in 10μl and dialyzed at 4°C for 16 hours in 100mM sodium phosphate pH 8.0, 150mM sodium chloride and 0.3% (w/v) CHAPS. The proteins were then reacted with the DSS reagent (1.2mM) for 2 hours at 4°C. Reactions were stopped by quenching with 1 volume of 100mM Tris-HCl pH 7.5 incubated on ice for 30 minutes. Proteins were separated on denaturing SDS-gradient gel (AnyKd, Bio-Rad). After electrophoresis, protein bands were detected with the Gelcode Blue dye. Myristoyltransferase reaction conditions are indicated in the legend of the figures and were performed as previously described [30]. Detection and quantification of the formation of the acyl-peptide in the presence of acyl-CoAs and the peptide GLYVSRLF was performed by reverse phase HPLC [30]. Calculations and statistical analysis were performed with GraphPad Prism 7.

### Reverse transcription and quantification

Total RNA was isolated with PureLink RNA Mini Kit according to the manufacturer instructions (ThermoFisherScientific). Purified RNAs were treated with RNase-free DNaseI (TURBO DNase, ThermoFisherScientific), extracted with acidic phenol/chloroform and concentrated by ethanol precipitation. DNA-free RNA was suspended in RNase-free water and stored at -80°C. Synthesis of cDNA was performed with the RevertAid First Strand cDNA Synthesis kit in the presence of random hexamer primers (ThermoFisherScientific). End-point RT-PCRs were performed with SuperScript^™^ One-Step RT-PCR System with Platinum^™^ Taq DNA Polymerase (ThermoFisherScientific). Real-time PCR reactions were performed with human *ACBD6*, *NMT1*, *NMT2* and *ACTB* gene specific PrimeTime qPCR Primers (IDT DNA), using iTAQ SYBR Green Supermix with ROX (Bio-Rad, Hercules, CA). Quadruplicated reactions were performed in 5μl volumes in a 384-well plate and were run on an ABI7900HT instrument (Applied Biosystems, Foster City, CA). Ct values of *ACBD6*, *NMT1* and *NMT2* were normalized to the values obtained for *ACTB*. Normalized values (ΔCt) obtained in control normal cells were used as reference and the ΔΔCt method was used to determine the fold change in expression of *ACBD6*, *NMT1* and *NMT2* in the mutant cells compared to the normal cells.

## Results

The roles of the acyl-CoA binding domain (ACB), ankyrin-repeat motifs (ANK) and of the linker region (Fig 1) in acyl-CoA binding, in stimulation of the activity of the human NMT2 enzyme and protection from palmitoyl-CoA were determined (see below).

### Acyl-CoA binding requirements of ACBD6

**Fig 2:**
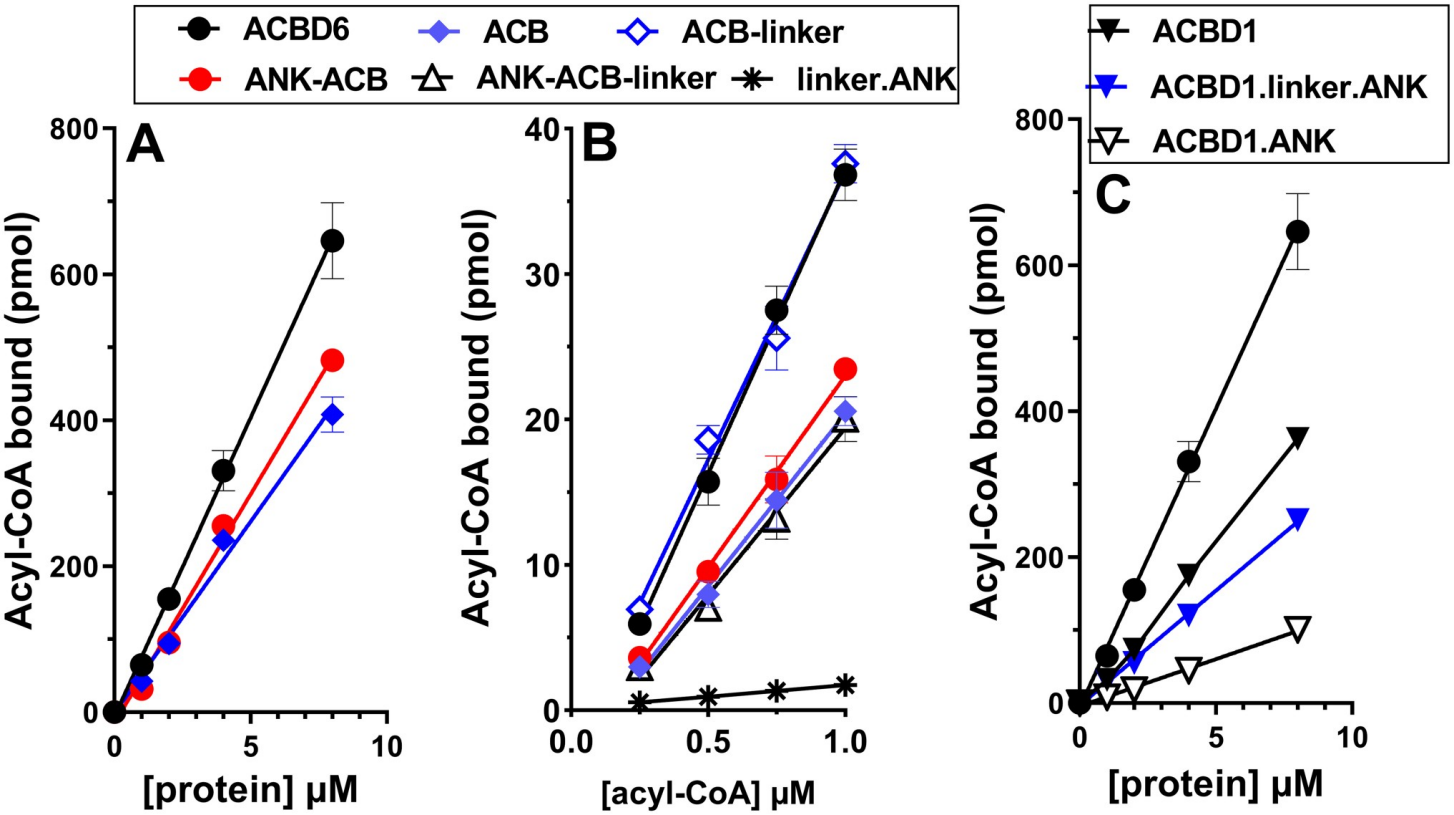
Analysis of module deletion and switch on acyl-CoA binding function. Binding assays were performed with 15μM ^14^C-C_18:1_-CoA and increasing concentrations of the indicated proteins (0 to 8μM) (Panel A and C) and with 2μM of the indicated proteins with increasing concentrations of ^14^C-C_18:1_-CoA (0.25 to 1μM) (Panel B). Error bars represent the standard deviations of values obtained from four measurements.

A recombinant form containing only the ANK module was insoluble [32] but it could be produced when fused to the linker region or to the ACB domain. The ACB domain is sufficient for acyl-CoA binding activity and it can bind even when the ANK module was switched to its amino-terminal end (ANK.ACB form) (Fig 2A). Fusion of the linker to ACB (ACB.linker construct) produced a form with a binding activity which was indistinguishable from the full-length protein (Fig 2B). However, fusion of the linker to the switched ANK.ACB form (ANK.ACB.linker) did not produce a form with as high binding capacity as ACB.linker. DBI (aka ACBD1) is the shortest member of the ACBD family and no functional motif appears present downstream of the ACB domain (Fig 1). Fusion of the ANK module of ACBD6 to ACBD1 produced a form (ACBD1.ANK) with reduced binding activity compared to ACBD1 (Fig 2C). Insertion of the linker in-between the two modules produced a form (ACBD1.linker.ANK) with greater binding activity. Thus, the linker is not directly involved in binding of acyl-CoA but might provide some flexibility for the independent folding of the two modules (see below).

### The C-terminal module protects NMT from competition

**Fig 3:**
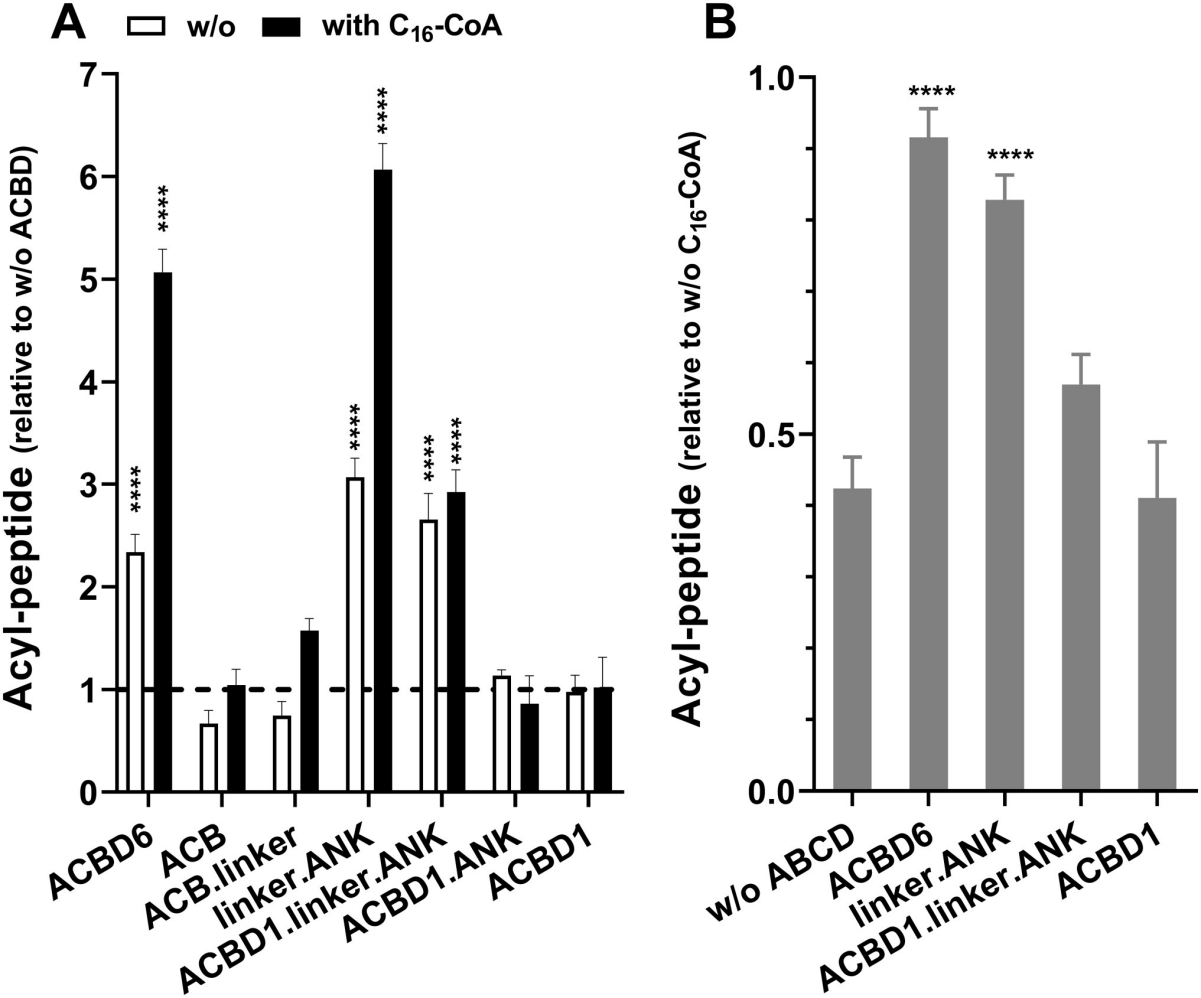
The C-terminal module is sufficient for NMT2 stimulation and protection. Formation of the myristoyl-peptide by NMT2 was measured in the presence of the indicated proteins over a concentration range of 0.016 to 4μM for 3 min at 37°C. The full data set is presented in S1 Fig. The bar graphs summarize the values obtained at 4μM. Human NMT2 enzyme was added at a concentration of 50nM in the presence of 5μM C_14_-CoA and in the absence (white bars) or presence of excess competitor C_16_-CoA (50μM) (filled bars). Control reactions were performed in the absence of the ACBD constructs and of C_16_-CoA. The data are presented relative to the value obtained in the absence of ACBD (panel A) or in the absence of C_16_-CoA (panel B). Error bars represent the standard deviations of values obtained from three reactions. **** indicate a *p* value of <0.0001.

**Fig 4:**
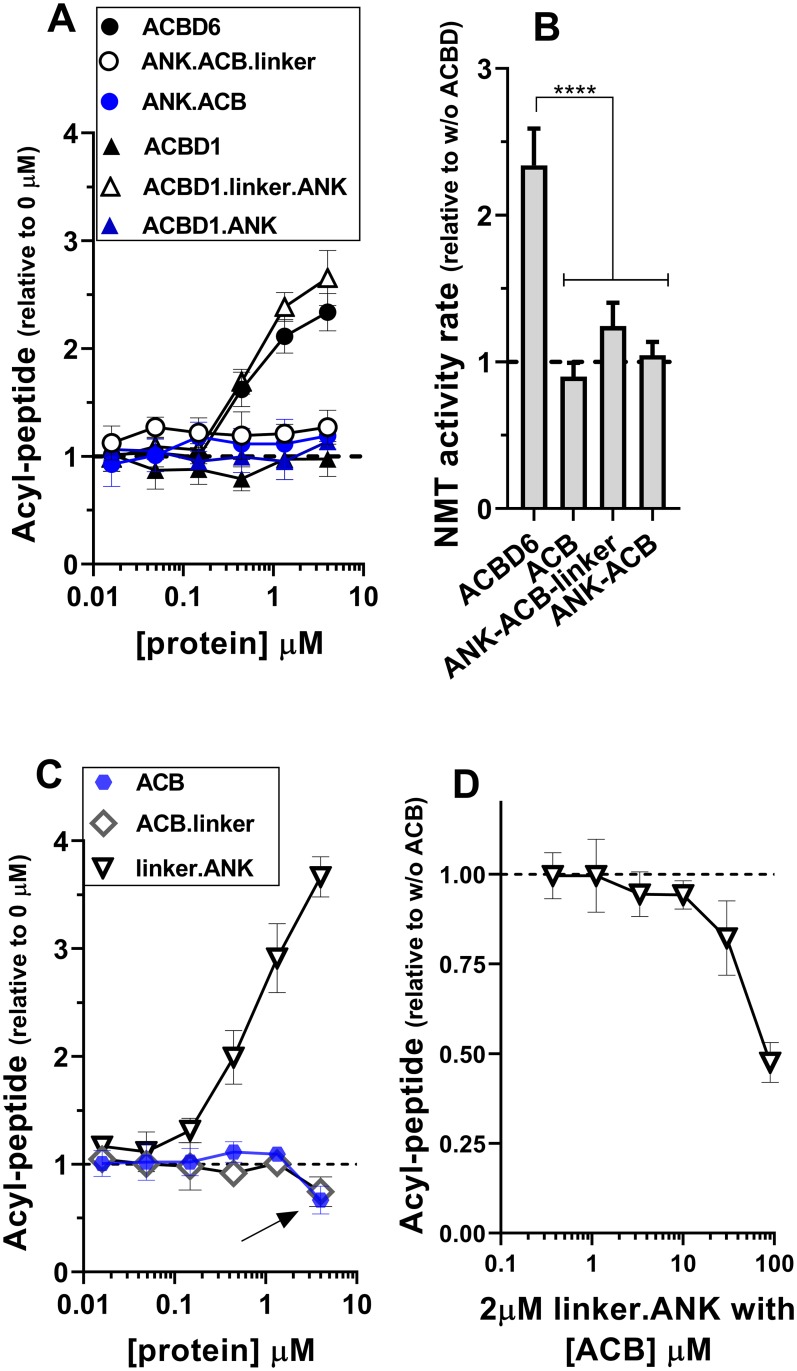
Role of the ACB domain and linker region in NMT2 activity. Formation of the myristoyl-peptide by NMT2 was measured in the presence of the indicated proteins over a concentration range of 0.016 to 4μM (panel A and C) and with 2μM of the linker.ANK form in the presence of increasing concentration of the ACB form (0.4 to 90μM) (panel D). Activity of NMT2 was measured in the presence of 4μM of the indicated proteins from 0 to 8 min and the rates of formation of the acyl-peptide are reported relative to the values obtained in their absence (panel B). Control reactions were performed in the absence of the ACBD constructs (panel A, C) and in the absence of ACB (panel D). The data are presented relative to the values obtained in their absence. The decrease in acyl-peptide formation in the presence of the ACB and ACB.linker forms observed at 4μM is indicated with an arrow in panel C. Human NMT2 enzyme was added at a concentration of 50nM in the presence of 5μM C_14_-CoA. Error bars represent the standard deviations of values obtained from three reactions. **** indicate a *p* value of <0.0001.

ACBD6 stimulates myristoyl-peptide formation and protects the activity of the human NMT2 and protozoan *Plasmodium* NMT enzymes from competition by other acyl-CoAs (Fig 3A) [29,30]. The ANK module is not necessary for acyl-CoA binding (Fig 2) but all the recombinant forms lacking this module failed to stimulate and to protect NMT (Fig 3A). Neither the ACB module nor the fully active acyl-CoA binding form, ACB.linker, could protect NMT2 (Figs 3 and 4). Acyl-CoA was not necessary and the linker.ANK form was as efficient as full-length ACBD6 in stimulating and protecting NMT2 (Figs 3 and 4C). Although, ACBD6 can supply the substrate (C_14_-CoA) and sequester the competitors (C_16_-CoA and C_12_-CoA) [29,30], the property of the linker.ANK form established that binding of the substrate and competitor are not enough to account for the effect of ACBD6. This was confirmed by the finding that even under condition resulting in a 10-fold molar excess of C_16_-CoA to ACBD6 (50μM vs. 4μM) and of C_16_-CoA to C_14_-CoA (50μM vs. 5μM), the NMT2 reaction was restored by ACBD6 and by the linker.ANK form to similar levels obtained in absence of the competitor (with to without ratio of 0.91 and 0.83, respectively; Fig 3B).

**Fig 5:**
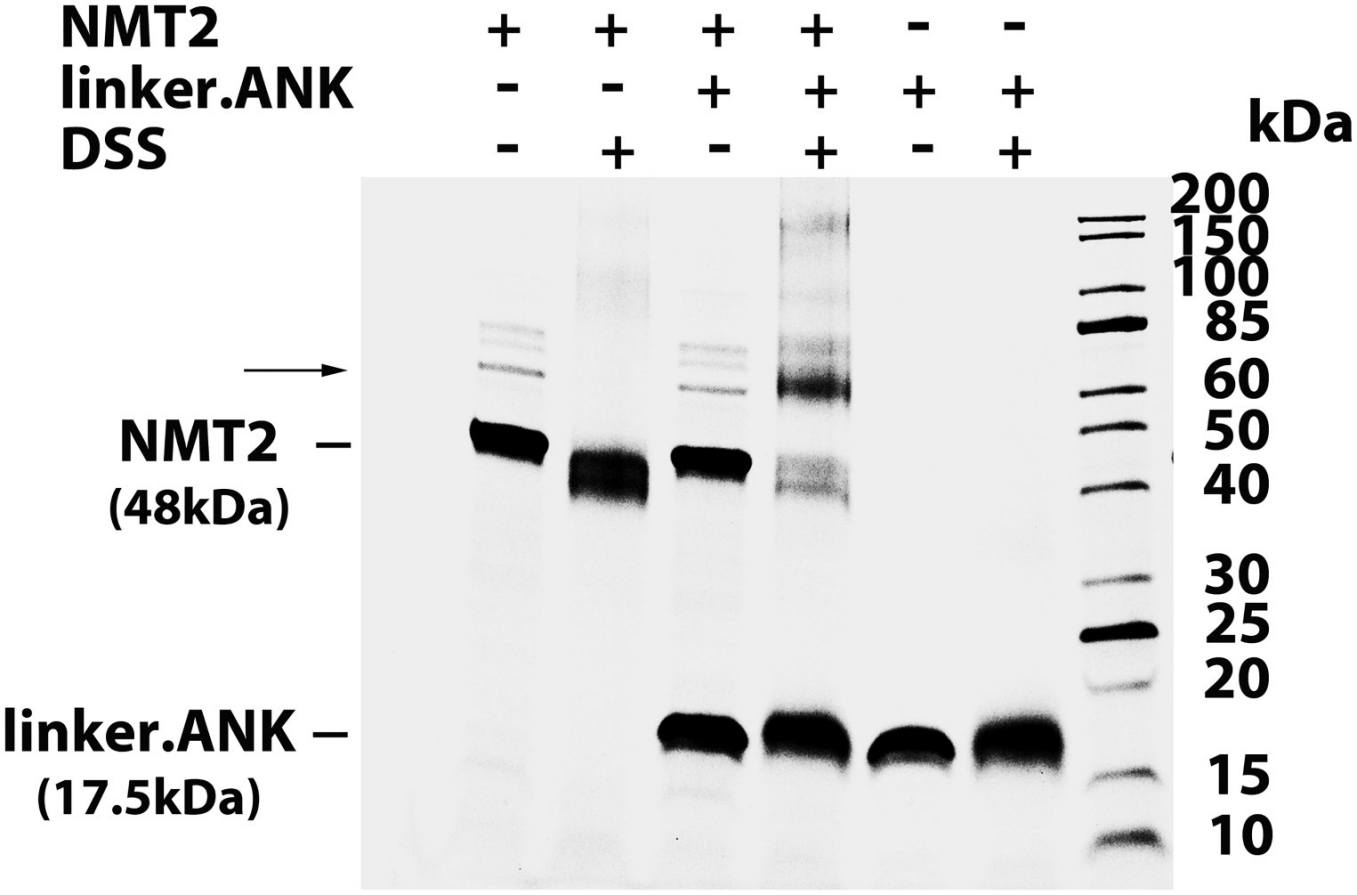
The C-terminal module is sufficient for NMT2 interaction. NMT2 (10μM) and the linker.ANK (30μM) recombinant protein were dialyzed together at 4°C for 16 hours. As indicated, the proteins were then reacted with the DSS reagent (1.2mM) for 2 hours at 4°C. Reactions were stopped by quenching with a solution of 100mM Tris-HCl pH 7.5. Proteins were separated on denaturing SDS-gradient gel (Any Kd, Bio-Rad). After electrophoresis, gel was stained with Gelcode Blue (Fisher Scientific). The panel shows the image of the gel with the position of NMT2 (48kDa), of linker.ANK (17.5kDa), and of a complex formed after treatment by DSS indicated on the left. The molecular masses of the ladder (Unstained Protein Standard, Broad Range; NewEnglandBiolabs) are shown on the right.

Formation of the ACBD6 / NMT2 complex can be detected following stabilization with a cross-linking agent [29]. Interaction of the fully active linker.ANK form with NMT2 was confirmed (Fig 5). In addition, the fusion of linker.ANK to ACBD1, which does not control NMT2, bestowed the stimulatory property of ACBD6 to the chimeric ACBD1 form (Figs 3A and 4A). The two switched forms (ANK.ACB and ANK.ACB.linker), which can bind acyl-CoA (Fig 2) and carry the ANK module required for NMT2 interaction, failed to stimulate NMT2 (Fig 4A and 4B). Similarly, the chimeric ACBD1.ANK form did not improve activity of NMT2 (Figs 3 and 4A). These findings confirmed the requirement of a linker region for the folding of the two modules. These findings also established that association to the ANK module is essential to regulate NMT2 activity and that it can act independently of the ACB module.

### Acyl-CoA binding to ACB enhances the stimulatory properties of ACBD6

In the presence of the competitor C_16_-CoA, the stimulatory effect of ACBD6 and linker.ANK was greater than in reactions performed in its absence. However, the stimulation provided by ACBD1.linker.ANK was not significantly different in the absence and presence of C_16_-CoA (Fig 3A and 3B). This was unexpected since an ACB module was not necessary to provide protection. The finding that the ANK domain, separated from the ACB of ACBD1 by the linker, was not able to act similarly than the form fused to the ACB of ACBD6 (that is, ACBD6) suggested an interference of the ACB domain of ACBD1 in the function of the downstream module. We observed that at high concentration, the ACB module of ACBD6 became inhibitory on NMT2 activity. In reactions performed with 5μM C_14_-CoA, the ACB and ACB.linker forms decreased NMT2 activity when their concentration reached 4μM (Figs 3A and 4C). The sequestration of the substrate by the isolated ACB domain was confirmed when the active linker.ANK form was incubated in the presence of increasing concentrations of the ACB form (Fig 4D). These findings provided further evidences that although, the acyl-CoA binding domain is dispensable, it plays a role in the ACBD6-dependent activity of NMT2. Differences in binding affinity for the substrate and competitor of the different ACBD members might prevent the substitution of the ACB domain. The ACBD1.linker.ANK chimera was successful in stimulating usage of C_14_-CoA by NMT but failed to fully enhance the myristoyltransferase reaction in the presence of C_16_-CoA.

### Role of the ligand in ACBD6-dependent NMT2 activation

**Fig 6:**
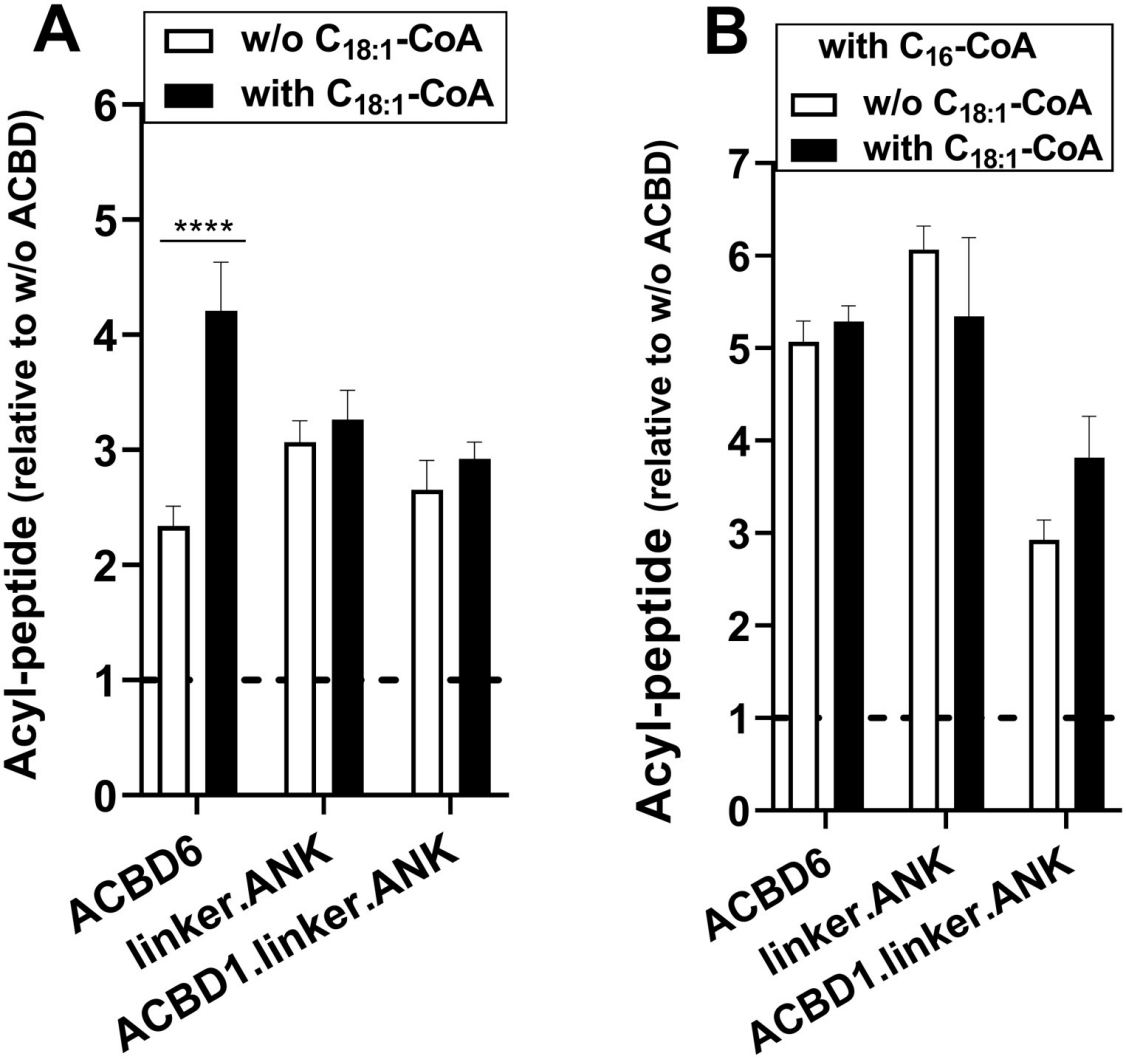
Acyl-CoA binding to the ACB domain enhances NMT2 activity. Formation of the myristoyl-peptide by NMT2 (50nM) was measured in the presence of the indicated proteins (4μM) and with 5μM C_14_-CoA. As indicated, C_16_-CoA (50μM) and C_18:1_-CoA (20μM) were added in the reactions. Control reactions were performed in the absence of the ACBD constructs and the data are presented relative to the values obtained in their absence. Error bars represent the standard deviations of values obtained from three reactions. **** indicate a *p* value of <0.0001. Differences detected with the linker.ANK and ACBD1.linker.ANK forms did not reach statistical significance of *p* ≤0.05.

Although, interaction of the ANK module to NMT2 is sufficient to stimulate and protect NMT2, when the ACB of ACBD6 is present, the substrate and the competitor will bind the ACB module. We have previously established that C_14_-CoA-bound•ACBD6 was a substrate of NMT2 and that sequestration of the competitor (C_16_-CoA-bound•ACBD6) prevented the de-esterification of C_16_-CoA and the irreversible blockage of the enzyme by the C16 acyl chain [29,30]. Compared to other acyl-CoAs, the substrate C_14_-CoA is a minor species and the more abundant competitor C_16_-CoA binds ACBD6 with the lowest affinity [33]. Hence, the presence of a complex mixture of acyl-CoAs and perhaps fatty acids *in vivo*, might interfere with substrate and competitor binding to both NMT2 and ACBD6 [25,27,30,33]. To mimic these conditions, reactions were performed with low substrate concentration (5μM), excess competitor (50μM) in the presence of a third acyl-CoA (C_18:1_-CoA, 20μM). C_18:1_-CoA is neither a substrate nor a competitor of NMT2 and binds with high affinity to ACBD6 [29,33]. In the absence of the competitor, the presence of C_18:1_-CoA resulted in the enhancement of the stimulatory property of ACBD6 (Fig 6A). Binding of C_18:1_-CoA to the ACB module of ACBD6 was required since the stimulation produced by the truncated linker.ANK form and the chimeric ACBD1.linker.ANK form were not enhanced by the presence of C_18:1_-CoA. In the presence of the competitor, the ACBD6-dependent NMT2 activity was not reduced in the presence of C_18:1_-CoA (Fig 6B). Given the binding property of ACBD6, neither the substrate nor the competitor could have displaced C_18:1_-CoA from the ACB module [33]. These findings provided further evidences that stimulation and protection of NMT do not rely on binding of the substrate and of the competitor to the ACB module. However, the ACB domain modulates the function of the downstream module since C_18:1_-CoA-bound•ACBD6 was more stimulatory than C_14_-CoA-bound•ACBD6 and, was as protective as C_16_-CoA-bound•ACBD6 (see Discussion).

### *ACBD6* loss-of-function mutations detected in individuals with NDD

**Fig 7:**
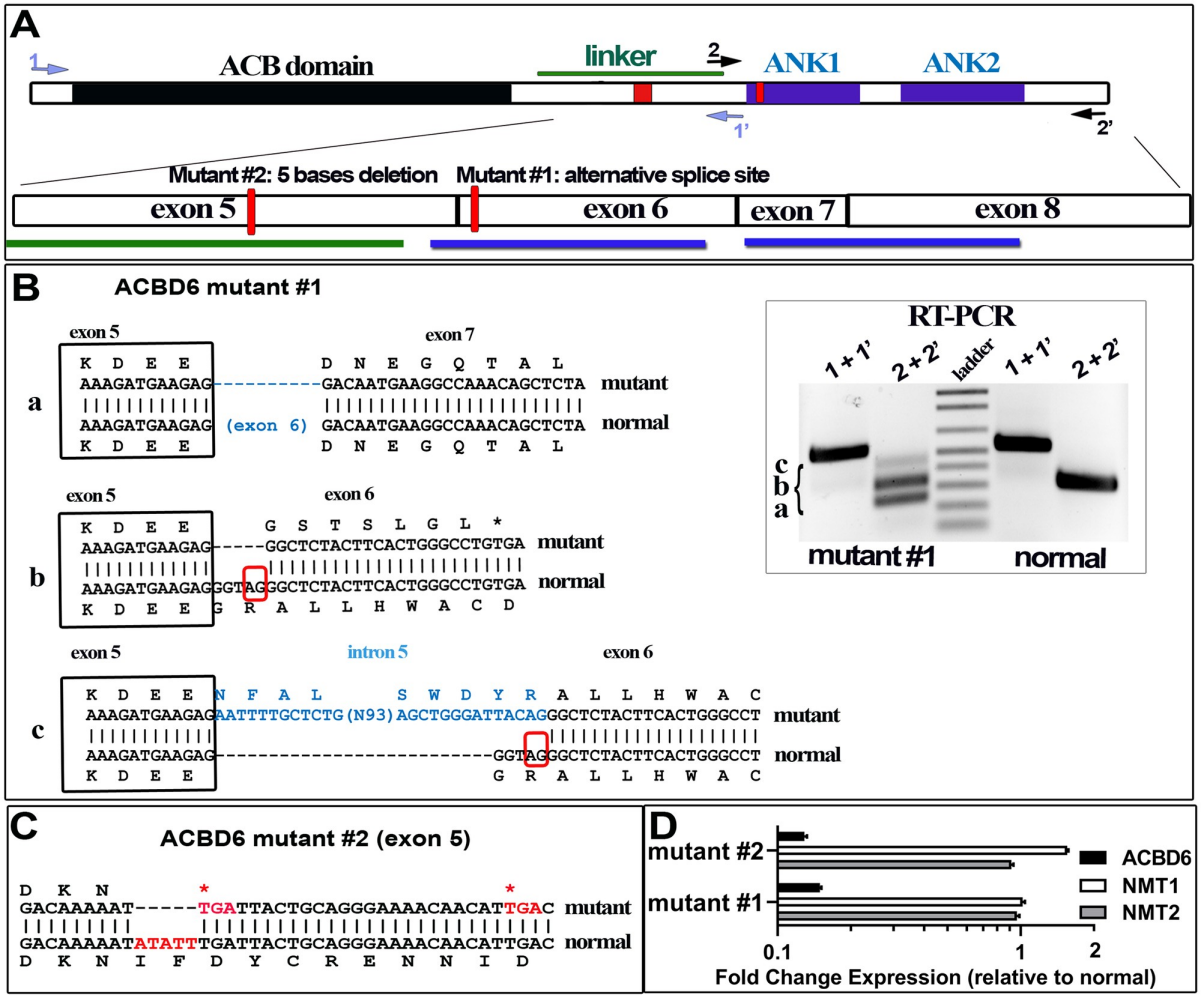
Spliced isoforms identification of human *ACBD6*-deficient fibroblasts. **Panel A**. The usage of an alternative splice site in exon 6 (mutant #1) and the 5 bases deletion in exon 5 (mutant #2) are indicated as red boxes on the cartoon representation of ACBD6. The exon organization of the linker and the ANK-repeat motifs (ANK1 and ANK2) region is also shown. The positions of the two primer pairs (1/1’ and 2/2’) used for reversed transcription of *ACBD6* mRNA are indicated. **Panel B**. Three cDNAs were identified with the RNA isolated from mutant #1 and are indicated as isoform a, b, and c. Separation of the cDNAs on 1.2% agarose gel is shown in the inset. Isoform a lacks exon 6 encoding the ANK1 motif; isoform b represents the splicing of exon 5 to the alternative splice acceptor site identified in exon 6, and result in the early translational stop removing the ANK1 and ANK2 motifs; isoform c is similar to isoform b but with an insertion of a sequence present in intron 5, and resulting in the disruption of ANK1. Because of the low level of expression of *ACBD6* mRNA in those cells, the very low abundant isoform c might represent an immature pre-RNA of isoform b detected by end-point RT-PCR. **Panel C**. The five bases deletion affecting exon 5 of *ACBD6* in mutant #2 is highlighted in red. The single isoform detected in these cells will produce a form truncated of the linker and ANK motifs. **Panel D**. Expression of *ACBD6*, *NMT1* and *NMT2* were determined by qRT-PCR, using *ACTB* as the reference mRNA. The values obtained with RNA isolated from the two mutant cells are reported relative to the values obtained with normal fibroblast. Note the logarithmic scale of the x axis. Error bars represent the standard deviations of values obtained from 4 measurements.

Mutations disrupting the coding sequence of the *ACBD6* gene were identified in two unrelated individuals with neurodevelopmental disorder (see Methods). Skin-derived fibroblasts were obtained and grown for further analysis (cell lines are referred as mutant #1 and #2; Fig 7A). A single base change (T>C) disrupts the splice acceptor site in intron 5 (rs765369140) of the *ACBD6* gene and results in the production of three spliced isoforms in mutant #1 (Fig 7B). These isoforms were not detected in the non-carrier sibling. The two most abundant cDNAs represent splicing events leading to slippage of exon 6 (isoform a) and of splicing of exon 5 to an alternative splice acceptor site (AG^578^) in exon 6 (isoform b). These two spliced mRNAs would lead to the production of forms truncated of ANK1 and of the two ANK-repeat motifs, respectively. A third cDNA of very low abundance, isoform c, represented the insertion of a sequence of 118 bases present in intron 5 at the alternative splice acceptor site of exon 6 (AG^578^) and disrupting the ANK1 motif. Whole exome sequencing of patient #2 revealed two homozygous variants located in the same autozygosity region on chromosome 1q25 in both affected brothers: *ACBD6* gene c.484_488del; p.(Ile162*), *PRDX6* gene c.136del; p.(ValCysfs*23). Both variants were confirmed by Sanger sequencing and segregated in the family. Both parents were heterozygous carriers of the detected variants. The 5-bases deletion in exon 5 of *ACBD6* results in the production of an isoform missing part of the linker region and is truncated of the ANK module (Fig 7C). In addition of lacking a normal ACBD6 protein, *ACBD6* mRNA levels were also reduced more than 20-fold compared to non-carrier fibroblast (Fig 7D).

### Increased sensitivity of *ACBD6*-deficient fibroblasts to NMT substrate competitor

**Fig 8:**
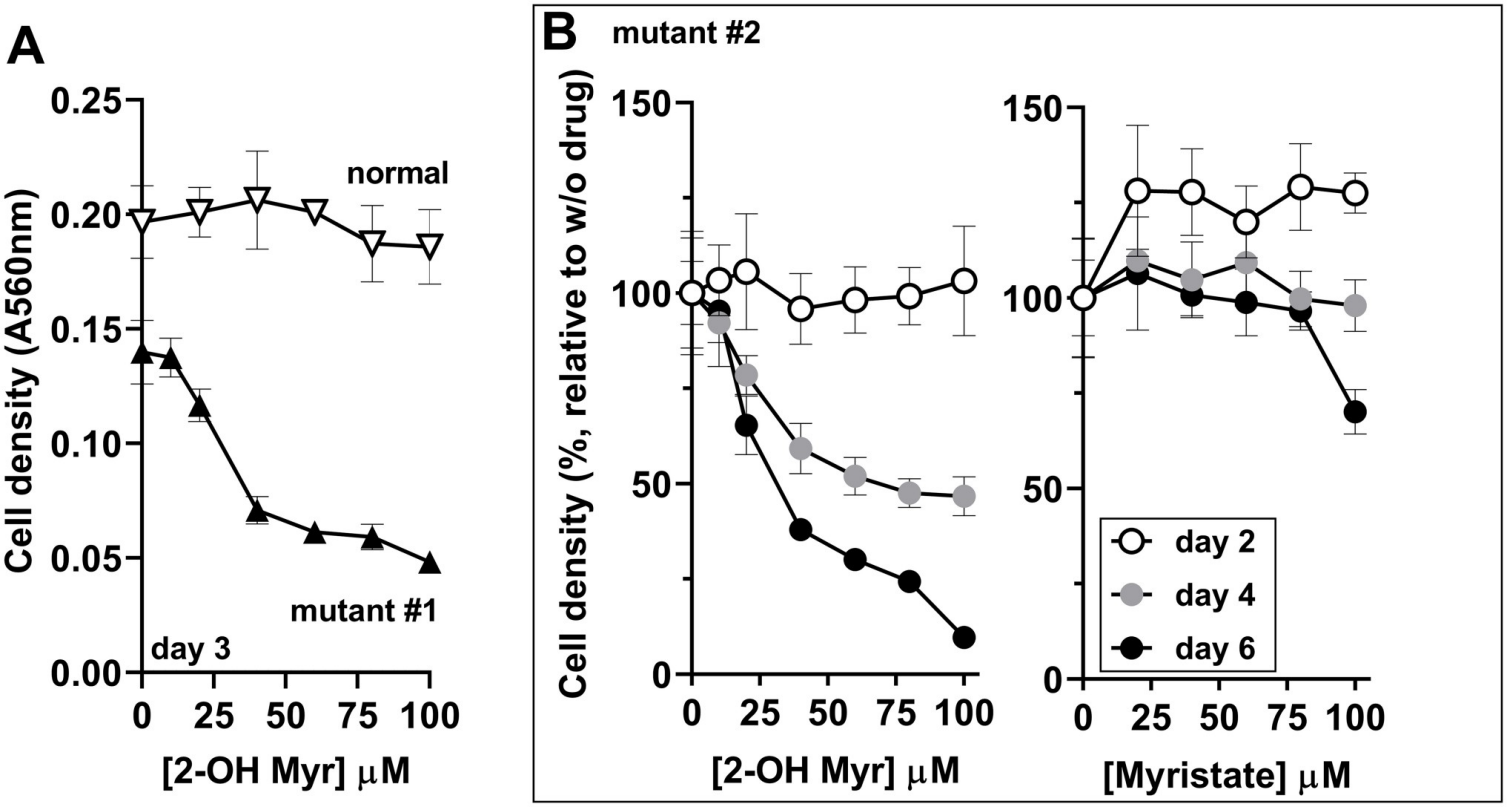
Decreased protection of NMT in *ACBD6*-deficient cells. Fibroblasts were grown in 96-well plates and exposed to the indicated concentration of 2-hydroxymyristate (2-OH Myr) and myristate. At the indicated times, the medium was removed and cells were fixed in 10% ice-cold TCA, stained with the SRB dye and absorbance was read at 560nm. In panel B, values obtained in the presence of the drugs are reported relative to the values obtained in their absence. Error bars represent the standard deviations of values obtained from 4 measurements.

The fatty acid 2-hydroxy myristic acid (2-OH Myr) is esterified to 2-hydroxy myristoyl-CoA (2-OH Myr-CoA) upon uptake into the cells. This myristoyl-CoA analog binds and is processed back to 2-hydroxy myristate by the thioesterase activity of NMT. However, the hydroxyl-myristate chain is not a substrate for the acyl-transferase reaction and blocks the enzyme (*in vitro* K_i_ = 45nM) [34,35]. At concentrations of ≥500μM, addition of 2-OH Myr in the culture medium results in the inhibition of NMT *in vivo* [34,36]. Concentrations of up to 100μM had little effect on the growth of the non-carrier fibroblast (Fig 8A). However, a dose-dependent growth inhibition by 2-OH Myr was observed with the *ACBD6*-mutant fibroblasts (Fig 8). Myristate was not inhibitory (Fig 8B and not shown). The expression of *NMT1* and *NMT2* was similar in the mutant cells compared to normal (Fig 7D). In confirmation of the protective property of ACBD6 observed *in vitro* in the presence of the palmitoyl-CoA (Fig 3), the increased sensitivity of the *ACBD6*-deficient fibroblasts to 2-OH Myr established that in the absence of ACBD6, the cells lack a mechanism to prevent NMT inhibition when challenged with a myristoyl-CoA binding competitor.

### Decreased myristoylation in *ACBD6* mutant fibroblasts

**Fig 9:**
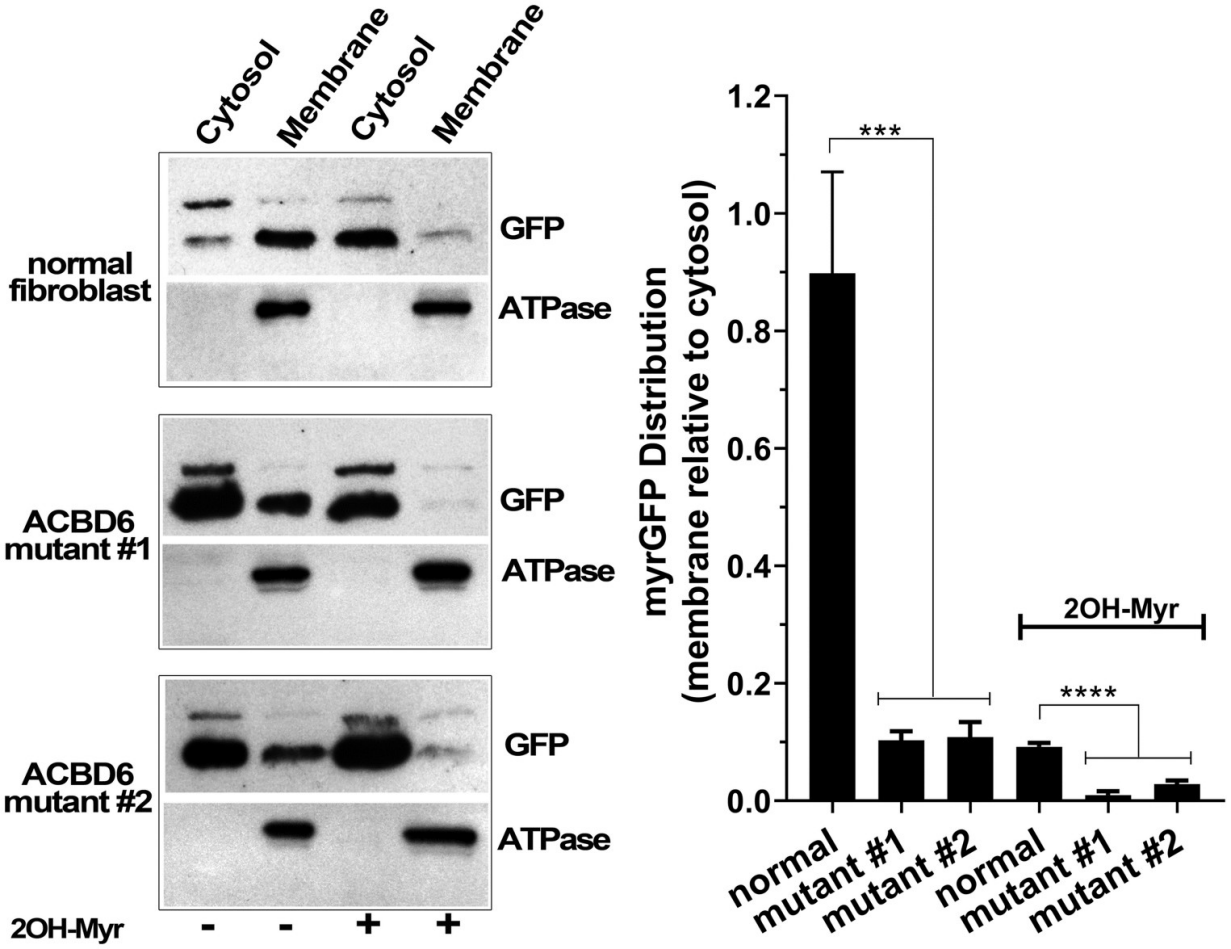
Decreased membrane localization of myrGFP in *ACBD6*-deficient cells. Fibroblasts were transfected with a vector expressing a myrGFP construct and grown for 48 hours in the absence or the presence of 100μM 2-OH Myr. The mRNA levels of NMT1 and NMT2 were not affected by such treatment and were similar to those of untreated cells. Cells were harvested, lyzed and the cytosolic and membranes fractions were isolated. Proteins were separated on denaturing SDS-PAGE, transferred to PVDF membranes, and GFP and the membrane marker Na+/K+-ATPase α1 were detected with mouse monoclonal antibodies. The bar graph shows the quantification of the signals of GFP in the membranes reported relative to the values obtained with the cytosolic fraction. Error bars represent the standard deviations of values obtained from three immuno-detection. *** and **** indicate a *p* value of 0.0001 and of <0.0001, respectively.

To confirm the deficiency in the N-myristoyltransferase activity of the *ACBD6*-mutant cells, the membrane distribution of GFP fused to the myristoylation signal of tyrosine kinase Src protein was analyzed [35,37,38]. Addition of a C14 aliphatic tail to soluble GFP confers the ability of myrGFP to associate with membranes. Cells were transfected with the recombinant construct and membranes were isolated (Fig 9). In the normal cells, approximately half of the produced myrGFP was enriched in the membrane fraction (membrane-to-cytosol ratio of 0.9 ±0.17). GFP (without the myr signal) was only detected in the cytosol (not shown). Incubation of the transfected cells with the NMT inhibitor 2-OH Myr resulted in a significant distribution shift from membranes to the cytosol (ratio of 0.09 ±0.01). The two *ACBD6*-deficient cell lines exhibit decreased membrane localization of the myrGFP construct, which is likely due to reduced myristoylation of the construct (ratio of ≈0.1 ±0.02) (Fig 9). The membrane association of myrGFP in the mutant cells was as low as in the inhibitor-treated normal cells (0.1 and 0.09, respectively). Challenge of the *ACBD6*-deficient cells with 2-OH Myr further decreased membrane association of myrGFP. Thus, in human cells, the absence of ACBD6 results in deficiency of the N-myristoylation of proteins and establishes the essential cellular function of this protein in supporting the activity of the NMT enzymes.

## Discussion

The ACB domain of ACBD6 is sufficient for acyl-CoA binding (short, medium, long and very-long acyl-CoA) [33] and the ANK domain is sufficient for interaction, stimulation and protection of NMT. The role of ACB in the functions carried by the different C-terminal domains of the members of the ACBD family is still poorly understood. The ACB domain of TgACBP2 is essential for growth of *Toxoplasma gondii* [39] and for the stimulation of the ceramide synthase activity of CerS2 and CerS3 by ACBD1 [40]. Interaction of peroxisomal ACBD5 with VAPB does not require the ACB domain [41] but a form truncated of the domain could not restore normal level of β–oxidation of very-long-chain fatty acids in an ACBD5 KO cell [42]. On the other hand, an ACBD3ΔACB form rescued the viral development defect of ACBD3 KO cells [43]. Although, the acyl-CoA bound to the ACB domain can be channeled to the enoyl-CoA isomerase domain of ACBD2, an ACB truncated form was active and carried the enoyl-CoA isomerase reaction in absence of acyl-CoA binding [44]. Thus, even for an ACBD member carrying an enzymatic activity with a requirement for acyl-CoA, the presence of an acyl-CoA bound to the ACB domain does not appear essential. The property of an ACBD6 form to enhance NMT only required the presence of the ANK module and indicates that the formation of a NMT2 / ACBD6 complex was sufficient. The property of human ACBD6 and of the ANK-containing ACBD protein of *Plasmodium falciparum* (PfACBD6) to interact and regulate the activity of the PfNMT enzyme confirms this conclusion [30].

Monitoring the thio-esterase and acyl-transferase activities of NMT in the presence of ACBD6 and feeding the reactions with a single acyl-CoA, with substrate and competitor, and with a mixture of acyl-CoAs indicate 3 properties of ACBD6: 1-supply, 2-sequestration, 3-allosteric activation. 1-The ACB domain binds C_14_-CoA and increases supply of the substrate to NMT. Other acyl-CoA binding proteins can minimally enhance activity of NMT but only ACBD6 can provide significant activity enhancement, even at low substrate concentration [30]. Phosphorylation of two serine residues in the ACB domain, which is unique to ACBD6, also enhanced the supply property of ACBD6 [30]. Fusion of the ANK module to ACBD1 resulted in a chimera with the enhancing supply property of ACBD6. 2- ACB binds C_16_-CoA and C_12_-CoA, which are two competitive inhibitors for NMT, and prevents their de-esterification to acyl chains that will occupy the myristoyl-CoA site and block the enzyme. This sequestration property is detected in absence of the substrate and ACB mutants affected in acyl-CoA binding failed to protect NMT [29,30]. The chimera ACBD1.linker.ANK was not as effective as ACBD6 in protecting NMT. Difference in binding properties of the different ACBD members might prevent the substitution of the ACB domain, and could also reflect the inhibitory potential of the ACB domain. The isolated ACB of ACBD6 was inhibitory by mean of substrate sequestration. Substrate and competitor bind to the ACB module [29,30] but the stimulatory and protective property of the ACBD6 form truncated of the ACB domain establish that supply and sequestration were not essential function of ACBD6. 3- Under *in vivo* conditions, ACBD6 will bind acyl-CoAs that are neither the substrate (minor species) nor the competitor (low affinity) and will not impede ACBD6-dependent NMT activity. In fact, ligands bound to ACB act as positive effector of the activation mechanism of NMT by ACBD6. The *increased* utilization of C_14_-CoA and the *persistence* of the protection from C_16_-CoA of NMT by the (C_18:1_-CoA)•ACBD6 form compared to (C_14_-CoA)•ACBD6 and to (C_16_-CoA)•ACBD6 suggest a mechanism controlling substrate/competitor access to the myristoyl-CoA binding site.

The formation of a complex with ACBD6 probably represents a protective mechanism of NMT enzymes to the challenge of specifically binding a very low abundant substrate in a mixture of acyl-CoAs. The first erroneous de-esterification of C_16_-CoA will likely irreversibly block the enzyme since the acyl-transferase step is highly specific of the C14 acyl chain [27]. The lack of specificity of NMT for the C14 acyl chain, observed with the purified enzyme, does not occur in the presence of ACBD6. Conformation changes induced by the binding of myristoyl-CoA are required to expose the peptide binding site, for the rapprochement of the glycine-peptide chain to the myristoyl chain, for the rotation of the Cα amine of the glycine residue, for the escape of CoA, and the release of the acylated-peptide chain [8] [45,46]. In this multi-steps process, the binding of myristoyl-CoA is not rate limiting. Thus, if the acyl-CoA competitor can be prevented from interfering with substrate binding, the myristoyltransferase reaction should proceed without hindrance. Interaction with ACBD6 could alter the conformation of the myristoyl-CoA site of NMT resulting in an increase specific for a C14 acyl chain and unable access of other acyl-CoAs. In this scenario, substrate/competitor binding to the ACB domain of ACBD6 would not be required and formation of the ACBD6/NMT complex would be sufficient to improve production of the myristoyl-peptide even in the presence of the competitor. Supply of C14 and sequestration of C16 chains by the ACB might not be essential but these two properties of ACBD6 likely improve the efficiency of the system *in vivo*. The positive effector property of ligands bound to the ACB might represent the first evidence for a role of acyl-CoA in the diverse cellular functions of members of the ACBD family. The enhanced property of ACBD6 induced by ligand binding and by phosphorylation of the ACB module might be essential to overcome interference by acyl-CoAs and fatty acids on the activity of the ACBD6/NMT complex. Altogether, these findings provide an explanation for the involvement of a member of the acyl-CoA binding family in supporting the activity of the myristoyl-CoA::N-acyl transferase enzymes.

We describe the functional relevance of homozygous *ACBD6* loss-of-function mutations. *ACBD6* mutations have not been associated with a disease so far. However, *ACBD6* gene is included in a genetic panel testing for mental retardation (https://www-ncbi-nlm-nih-gov.ucsf.idm.oclc.org/gtr/tests/558415/methodology/), with a potential association with autosomal recessive microcephaly [47], and several intron variations are associated with other clinical traits (https://www-ncbi-nlm-nih-gov.ucsf.idm.oclc.org/gap/phegeni?tab=1&gene=84320#pgForm). The T/C/A rs765369140 polymorphism affecting the splice acceptor site of intron 5 of *ACBD6* has been identified with frequency of 1/251302 (T>C)) and 1/121308 (T>A) (https://www-ncbi-nlm-nih-gov.ucsf.idm.oclc.org/snp/rs765369140). The *ACBD6* and *PRDX6* mutations detected in patient #2 were not listed in gnomAD, dbSNP, GoNL, HGVD, HGMD, or ClinVar. The *PRDX6* variant was classified as most likely benign, since no phenotype has been associated with this gene and mice homozygous for disruptions of this gene are anatomically normal, viable and capable of reproduction [48]. The single *ACBD6* mutation of patient #1 confirms that the loss-of-function of ACBD6 accounted for the N-myristoylation deficiency described in this work. The individuals are affected by intellectual disability which suggest a myristoylation deficiency in cells essential for brain functions. The diverse functions of regulators such as myristolated alanine rich C kinase substrate (MARKS), Fragile X mental retardation-related 2 protein and the neuronal Src isoform would be affected if the NMT enzymes were unable to assure their membrane association [15,19,49–51]. In these cells, the increased sensitivity to myristoyl-CoA competitor confirmed that in the absence of *ACBD6*, cells lack a mechanism to protect the N-myristoyltransferase reaction. We propose that in human cells, ACBD6 is essential for the N-myristoylation modification of proteins and that the active *in vivo* form of the NMT enzyme is defined by an ACBD6 / NMT heterocomplex.

## Supporting information

S1 Fig Myristoyl-peptide formation by NMT2 in presence of ACBD6 truncated, switched and chimeric forms. Formation of the myristoyl-peptide by NMT2 was measured in the presence of the indicated proteins over a concentration range of 0.016 to 4μM in the absence or presence of the competitor C_16_-CoA (50μM). Human NMT2 enzyme was added at a concentration of 50nM in the presence of limiting substrate concentration (5μM C_14_-CoA). Error bars represent the standard deviations of values obtained from three reactions.
(TIF)

S1 Raw images (PDF)
