# Correction: Requirement of the acyl-CoA carrier ACBD6 in myristoylation of proteins: Activation by ligand binding and protein interaction

**DOI:** 10.1371/journal.pone.0267678

**Published:** 2022-04-21

**Authors:** Eric Soupene, Ulrich A. Schatz, Sabine Rudnik-Schöneborn, Frans A. Kuypers

Following the publication of this article [1], it was noted that not all relevant data are within the manuscript and its Supporting Information files as stated in the Data Availability statement.

The underlying data for Figs 2–4, 6–9 and S1 are in S1–S17 Files. The cDNA sequence data, accession numbers OM258702, OM258703, OM258704 and OM258705, can be found on GenBank. With this correction, all relevant data are now provided.

The Data Availability statement is therefore updated to read: All relevant data are within the manuscript and its Supporting Information files. The cDNA sequence data can be found on GenBank under accession numbers OM258702, OM258703, OM258704 and OM258705.

In Fig 9 [1], the GFP and ATPase blot images included for the mutants are from different experimental replicates. The GFP and ATPase blot data in the S1 Raw Images file published with the article are from the same internal experiment; the ATPase image in the S1 Raw Images file is that shown in the figure.

## Supporting information

S1 File Underlying quantitative data supporting Fig 2A. (XLSX)

S2 File Underlying quantitative data supporting Fig 2B. (XLSX)

S3 File Underlying quantitative data supporting Fig 2C. (XLSX)

S4 File Underlying quantitative data supporting Figs 3A and 3B. (XLSX)

S5 File Underlying quantitative data supporting Fig 4A. (XLSX)

S6 File Underlying quantitative data supporting Fig 4B. (XLSX)

S7 File Underlying quantitative data supporting Fig 4C. (XLSX)

S8 File Underlying quantitative data supporting Fig 4D. (XLSX)

S9 File Underlying quantitative data supporting Fig 6A. (XLSX)

S10 File Underlying quantitative data supporting Fig 6B. (XLSX)

S11 File Original blot data supporting Fig 7B. (TIF)

S12 File Underlying quantitative data supporting Fig 7D. (XLSX)

S13 File Underlying quantitative data supporting Fig 8. (XLSX)

S14 File Original blot data supporting the ACB6 mutant #1 GFP blot in Fig 9. (TIF)

S15 File Original blot data supporting the ACB6 mutant #2 GFP blot in Fig 9. (TIF)

S16 File Underlying quantitative data supporting Fig 9. (XLSX)

S17 File Underlying quantitative data supporting S1 Fig. (XLSX)

## References

[1] Soupene E, Schatz UA, Rudnik-Schöneborn S, Kuypers FA (2020) Requirement of the acyl-CoA carrier ACBD6 in myristoylation of proteins: Activation by ligand binding and protein interaction. PLoS ONE 15(2): e0229718. 10.1371/journal.pone.0229718 32108178 PMC7046191

