# Supplementary figures and images for: Requirement of the acyl-CoA carrier ACBD6 in myristoylation of proteins: Activation by ligand binding and protein interaction
Source: PLoS One. 2020 Feb 27;15(2):e0229718. doi: 10.1371/journal.pone.0229718 (PMC7046191; doi:10.1371/journal.pone.0229718)

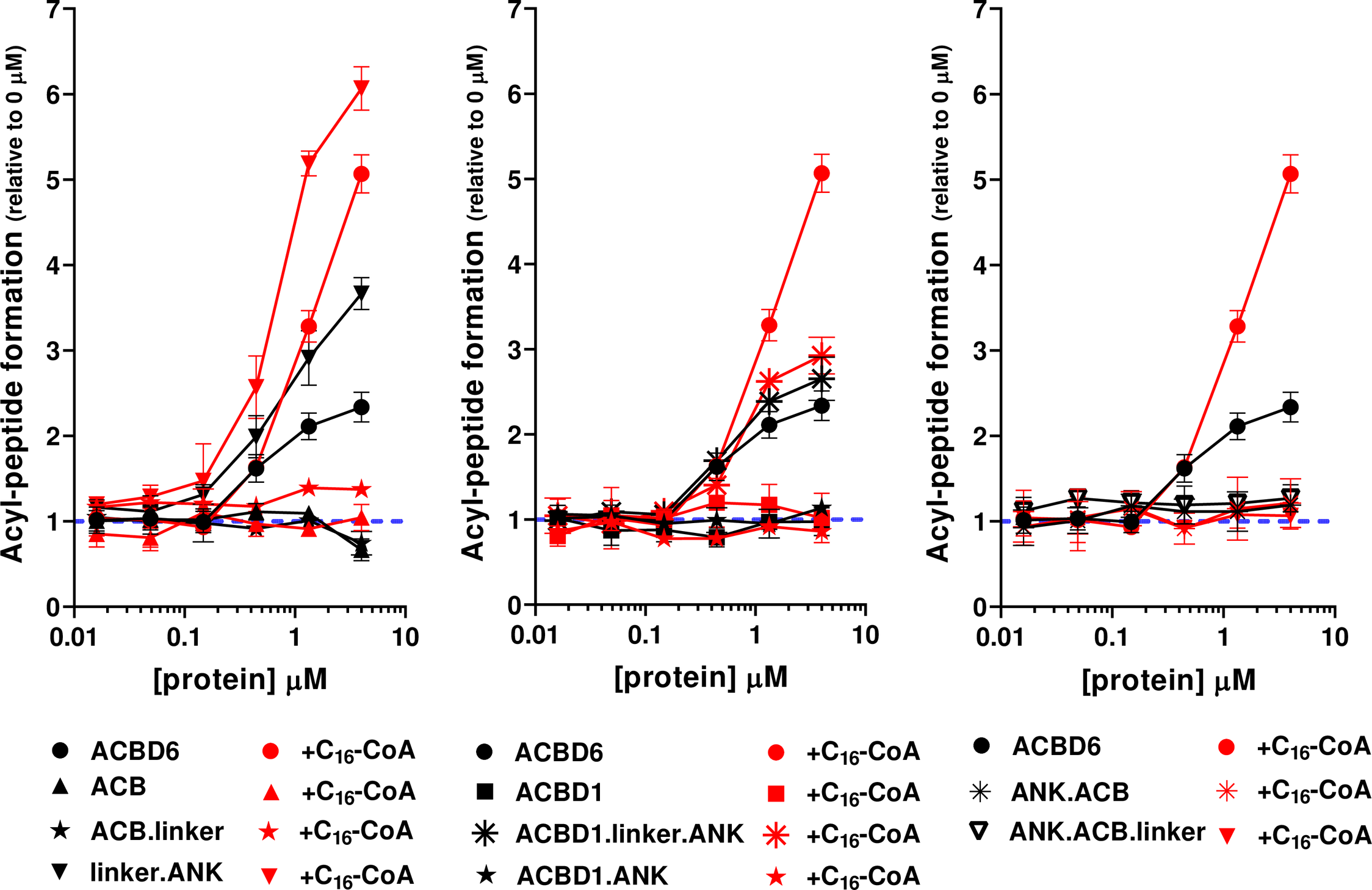

Supplement: S1 Fig — Formation of the myristoyl-peptide by NMT2 was measured in the presence of the indicated proteins over a concentration range of 0.016 to 4μM in the absence or presence of the competitor C16-CoA (50μM). Human NMT2 enzyme was added at a concentration of 50nM in the presence of limiting substrate concentration (5μM C14-CoA). Error bars represent the standard deviations of values obtained from three reactions. (TIF) [file pone.0229718.s001.tif]
