# Supplementary material for: Requirement of the acyl-CoA carrier ACBD6 in myristoylation of proteins: Activation by ligand binding and protein interaction
Source: PLoS One. 2020 Feb 27;15(2):e0229718. doi: 10.1371/journal.pone.0229718 (PMC7046191; doi:10.1371/journal.pone.0229718)

raw tif image for Fig.5 (coomassie-stained gel). AlphaImager camera

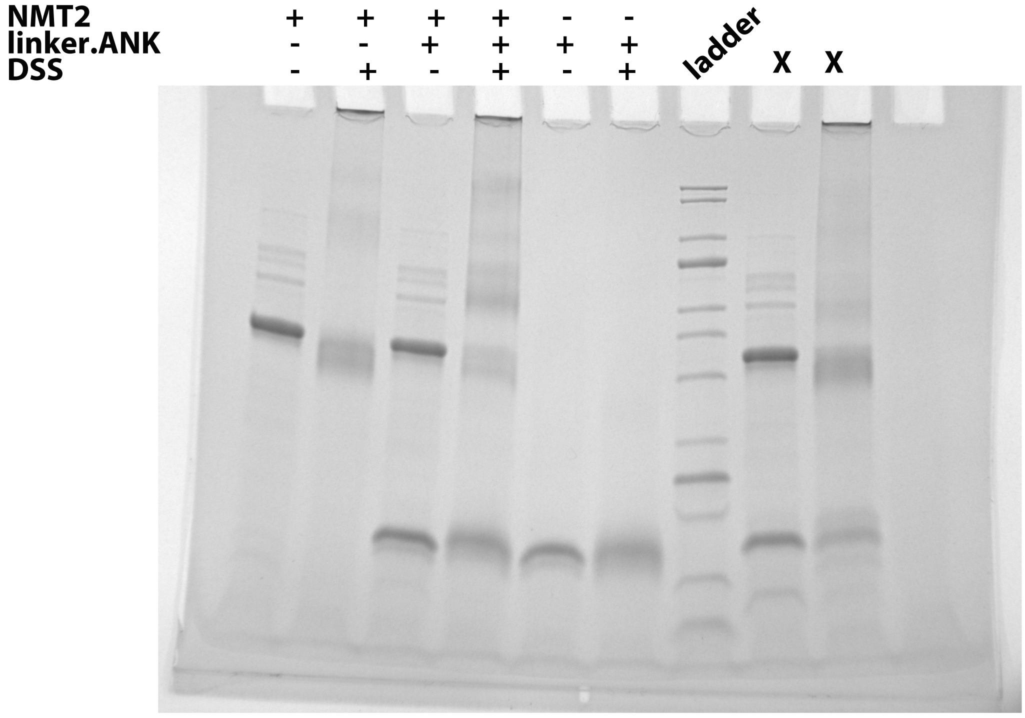

Raw tif images for Fig. 9: western-blot. AlphaImager camera
Top panels= GFP detection; Bottom panels=ATPase detection

Loading
1: Cytosol w/o 2OH-Myr
2: Membrane w/o 2OH-Myr
3: Cytosol with 2OH-Myr
4: Membrane with 2OH-Myr
5: Ladder

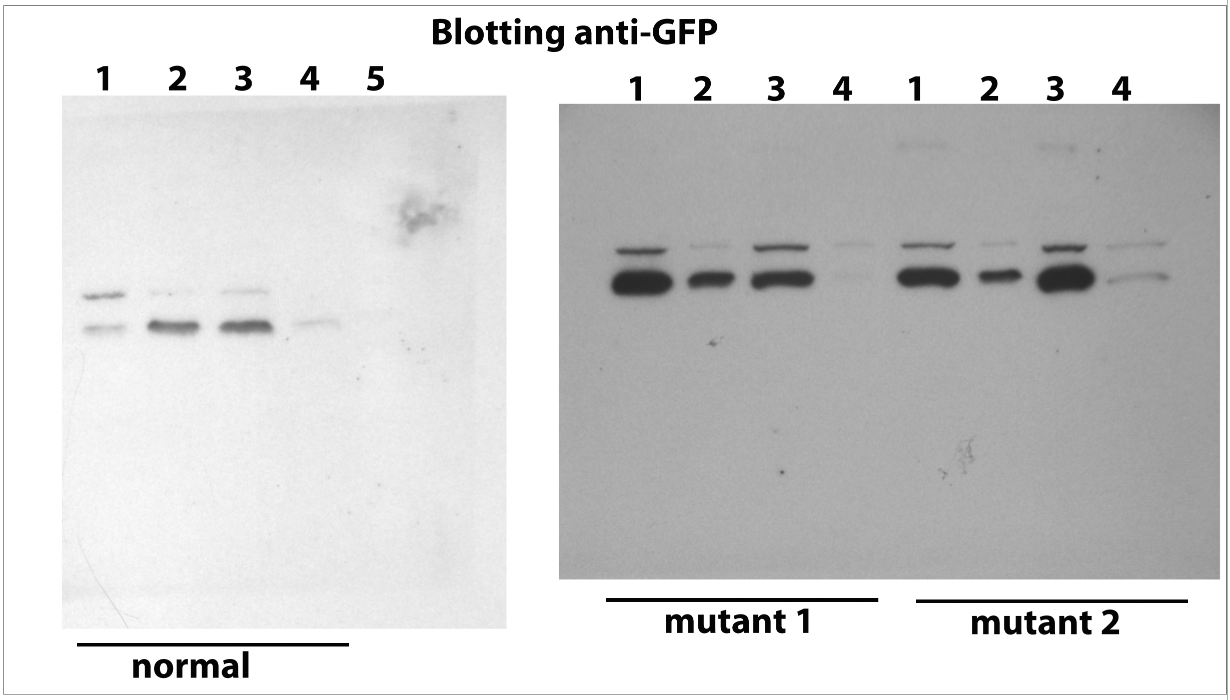

GFP-stained blots (above) were stripped (mild condition) and re-blotted with anti-ATPase (below)

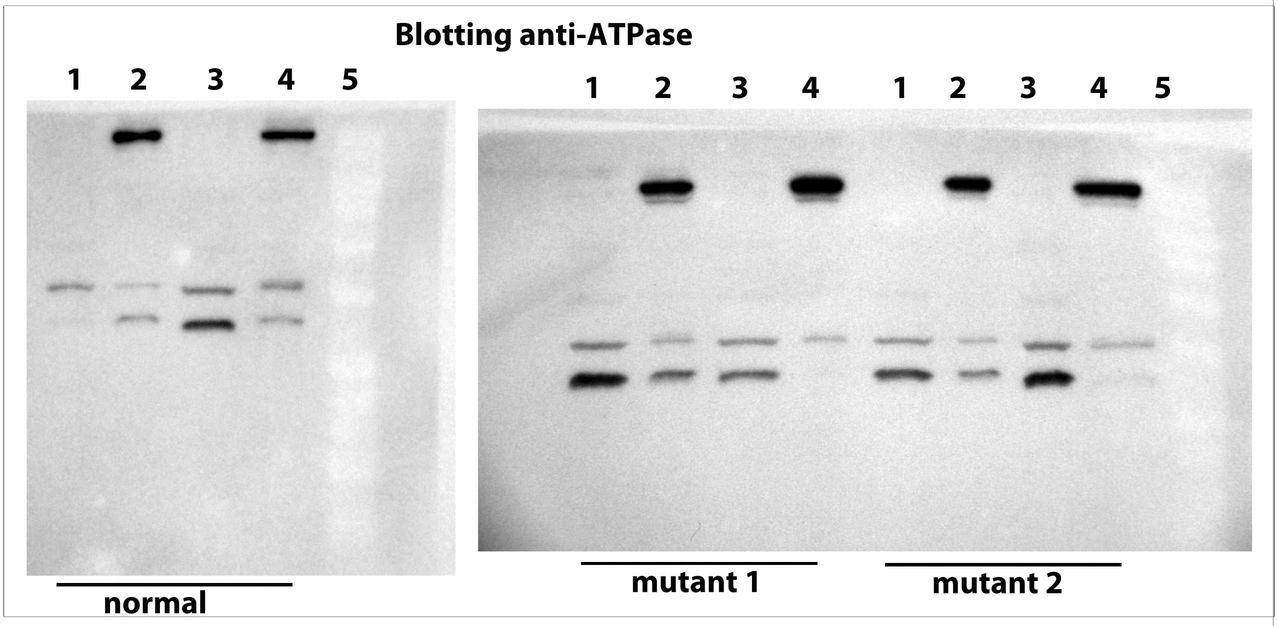

Supplement: S1 Raw images — (PDF) [file pone.0229718.s002.pdf]
